# Supplementary material for: Predictors for estimating subcortical EEG responses to continuous speech
Source: PLoS One. 2024 Feb 8;19(2):e0297826. doi: 10.1371/journal.pone.0297826 (PMC10852227; doi:10.1371/journal.pone.0297826)
Supplement: Attachment — Submitted filename: response_to_reviewers.pdf [file pone.0297826.s001.pdf]

We thank the reviewers for the constructive comments and have made several modifications following their suggestions, which we believe have improved the manuscript. Please find specific responses to reviewer questions below in blue text. Changes to the manuscript are in green text.

Reviewer 1

In this paper, the authors investigated the predictors for estimating the subcortical EEG response to continuous speech. This study provides a method to model and detect the subcortical processing of continuous speech. Even though this paper has some merits and is interesting, I still feel confused about several points that need to be addressed by the authors in details.

1. The abstract has not provided numerical results that make the audiences clear after glancing the abstract.

> We have now modified the abstract to give more quantitative details regarding the key results. The modified sentences are given below.
>
> "We also investigate the data length required for estimating subcortical TRFs, and find that around 12 minutes of data is sufficient for clear wave V peaks (> 3 dB SNR) to be seen in nearly all participants. Interestingly, predictors derived from simple filterbank-based models of the peripheral auditory system yield TRF wave V peak SNRs that are not significantly different from those estimated using a complex model of the auditory nerve, provided that the nonlinear effects of adaptation in the auditory system are appropriately modelled. Crucially, computing predictors from these simpler models is more than 50 times faster compared to the complex model."

2. Some important concepts are not clear and confusing. "Predictors" is what I concern most. TRF is recognized as predictor derived from the stimulus, but the authors also "compare predictors ... for estimating TRFs". Moreover, "rectified speech waveform as a predictor". Why the waveform can be predictor also? It is really confusing me. It is strongly suggested to clearly define the "predictor".

> We apologise for the confusion. We used the term 'predictor' to refer not to the TRF itself but rather to the input to the TRF model. This aligns with how the term 'predictor' is used in statistics to denote an independent variable in a regression problem, and this terminology has also been adopted in several previous studies that use TRFs in a similar context. The predictor could directly be the audio waveform (i.e., speech stimulus), or it could be a time-series that is considered to be relevant

to the neural processes under investigation. Often in linear TRF analyses, the predictor is constructed using some non-linear transformation of the audio stimulus (e.g., envelope, rectification) in order to account for the non-linearities of the early auditory system, and this predictor is then used to estimate linear TRFs. Therefore, the rectified speech waveform is indeed one of the predictors that is used in this work, along with the waveforms that are generated from the auditory models (GT, OSS, OSSA, ZIL). Finally, the predictor is different to the prediction, since the prediction is the 'output' of the TRF model (or the predicted dependent variable in a regression problem) and in our case corresponds to the EEG signal. In this work we use the prediction correlation (correlation between predicted and actual EEG) as a measure of model fit.

We have added the following sentences to the introduction for further clarification.

"The TRF is a linear model that relates the EEG signal to a stimulus-derived predictor, and therefore cannot capture the non-linear processing stages of the auditory system. However, the predictor, serving as the input to the TRF model, can be constructed to be a feature (or transformation) of the speech stimulus relevant to the auditory system. Accounting for peripheral non-linearities in the predictor could help 'linearize' the TRF estimation problem and lead to improved TRF models that reflect the activity of later neural processes."

3. Some details are missing. Which ear was given stimuli? How to prove the speech segments convey the same energy, or power (72 dB SPL), as the compared methods? From the spectrogram? It is said that the electrodes were placed on the mastoids and earlobes. What's the usage of the electrodes on earlobes? There is no information for this. How the speech stimuli were given to the subjects? In what manner? What are the parameters? What is the difference between Rectified Speech and the one in [5]? What does they look like? It is suggested to provide some figures to show the difference between RS, GT, OSS, OSSA, and ZIL.

We have now added a statement to the Methods section explaining that the stimulus was presented to both ears simultaneously (binaurally). We have added the following section to the Methods section to clarify how the speech stimulus was scaled to 72 dB SPL.

"The single channel speech segments were calibrated to be presented at 72 dB SPL using the following procedure: Speech shaped noise was generated by transforming white noise to have the long-term spectrum of the speech. This signal was then adjusted to be 72 dB SPL by recording the audio signals using a measurement amplifier (Bruel and Kjær Type

2636) and head-and-torso simulator (HATS, Bruel and Kjær Type 4128-C) containing two ear simulators (Bruel and Kjær Type 4158). The setup was calibrated using a sound source (Bruel and Kjær Type 4231). Each speech segment was then scaled digitally to have the same root mean square (r.m.s.) value as the 72 dB SPL speech shaped noise.

Additional electrodes were placed on the earlobes for a complete EEG montage, for future studies that may use the same dataset. However, in this work, we only use the Cz channel referenced to the mastoids as stated in the Methods, and do not use any of the other channels or electrodes. We have also mentioned in the Methods that the speech stimuli were presented using earphones in 8 short segments ($M_{duration}$ = 6 minutes 0 seconds, $SD_{duration}$ = 55 seconds). There were no other parameters used, apart from ensuring the signals were scaled to 72 dB SPL using the method outlined above.

The Rectified Speech predictor was generated in the same manner as given in [5] (Maddox 2018), using our speech stimuli, and this is now directly mentioned in the Methods. We have also added a new figure (Figure 1) that compares the predictors for a short speech segment as suggested by the reviewer. We prefer not to dedicate too much space in the manuscript to the predictors themselves, since other studies have analysed the outputs of these models and the focus of this work is not on the predictor waveforms but rather on the resulting TRFs. The new figure is replicated here for the reviewer's convenience.

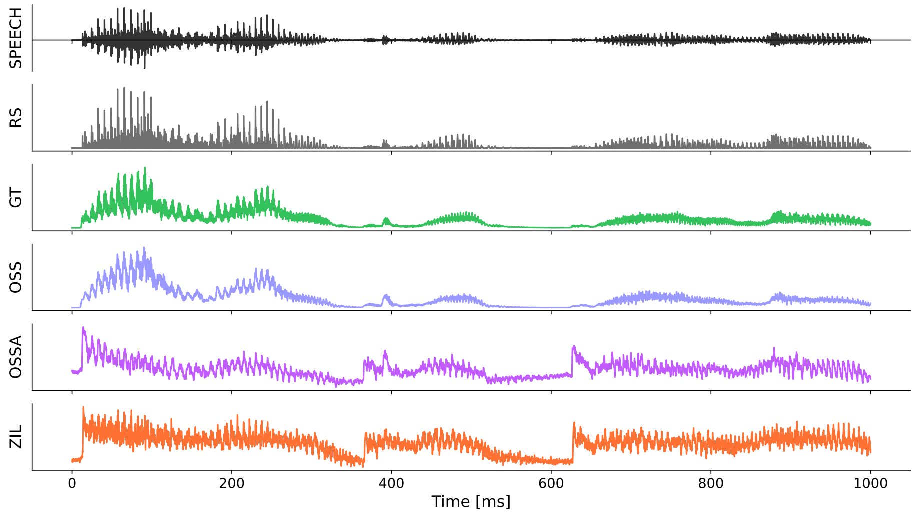

Figure 1. Predictor waveforms. The predictor waveforms are shown for a 1-second speech segment (also shown in the top row) to illustrate the

differences between the models. Note that the OSSA and ZIL predictors are the most different from the speech waveform, since they incorporate more peripheral non-linearities and adaptation effects.

4. More details should be provided for the model fit. Since this is an important metric for evaluation. Also, how these aforementioned methods derived from the auditory model should be clarified.

The model fits were calculated as the Pearson correlation between the predicted EEG signal and the actual EEG signal, with the predicted EEG in each case generated by the TRF model that was estimated using each of the auditory model predictors. We have now further expanded the relevant section in the Methods as follows, and this section provides all the details in model fit calculation. No other procedures were used when calculating the model fits.

"The goodness of fits of the TRF models were evaluated using prediction correlations. The average TRF across positive and negative predictors fit on the training dataset was used to predict the EEG signal of the test trial by convolving it with the appropriate predictors, and subsequently the Pearson correlation between the predicted EEG and the actual EEG signal was calculated. The correlations across all cross-validation folds were averaged together to form an estimate of the model fit. To estimate the noise floor, a null model was formed by averaging the prediction correlations from TRFs that were fit on circularly shifted predictors (shifts of 30, 60 and 90 seconds), similar to typical null models used in prior work with cortical TRFs (Kulasingham et. al., 2020). This method preserves the temporal structure of the stimulus, while destroying the alignment between the stimulus and the EEG, resulting in an estimate of the noise floor. The same leave-one-out cross-validation approach at each data length was followed for the null models."

5. I am wondering the cut-off frequency of highpass filter is appropriate or not. First, after filtering, is the speech sound naturally? Second, in PTA, we know that the 500 Hz was also tested for the human being. Also, in tone-burst ABR, 500 Hz could also induce highly recognized ABR signal. It is appropriate or not to use 1kHz highpass filter to process the audio should be investigated.

High-passing speech with a gentle 1 kHz filter results in natural sounding speech in which a lot of power between 125-1000 Hz as well as the pitch information is clearly preserved. As can be seen in the figure below, the filter enhances the relative contribution of higher frequencies. This method was also used in previous work to detect subcortical TRFs (Maddox and Lee 2018). Subcortical responses are more strongly driven by the high frequency content of speech stimuli (Abdala and Folsom 1995),

and therefore this highpass filtering should allow for better estimation of TRFs.

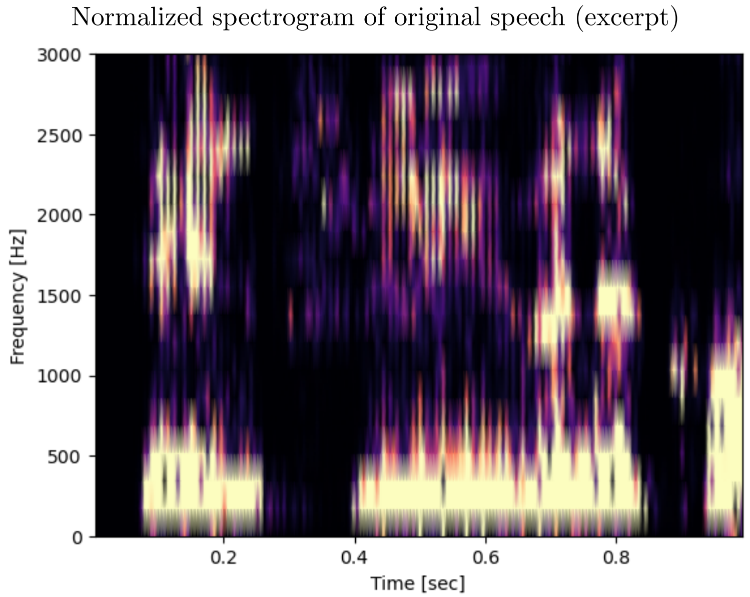

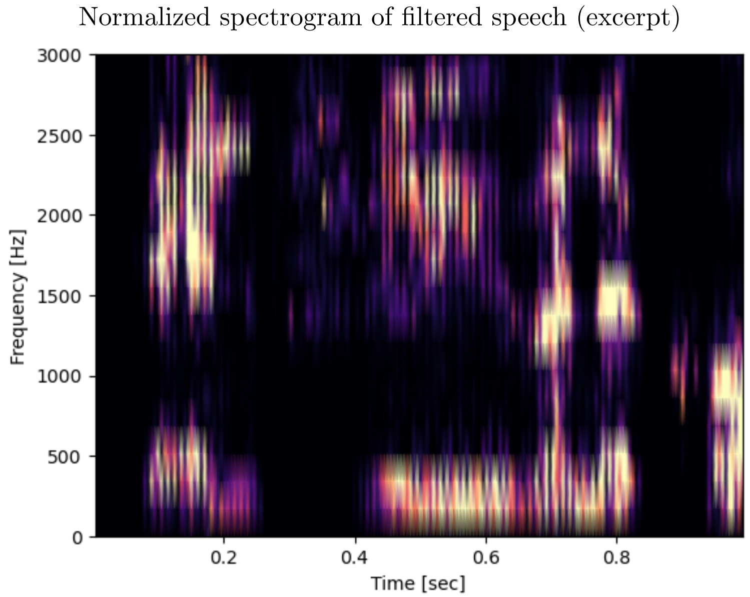

We have now expanded the relevant section of the Methods as follows.

"The 2-channel audio was averaged to form a mono audio channel, which was then highpass filtered at 1kHz using a first order Butterworth filter

to enhance the relative contribution of high frequencies, since the brainstem response is more strongly driven by high frequencies (Abdala and Folsom 1995). Using this gentle highpass filter resulted in natural sounding speech in which a lot of power between 125-1000 Hz as well as the pitch information is clearly preserved. This method was also used in prior studies to detect clear subcortical TRFs (Maddox and Lee 2018)"

6. Grounded metal box can eliminate the electromagnetic noise. But how can this avoid stimulus artifact? Please provide more details about this. For click, stimulus polarity alternation can avoid the stimulus artifact, how can speech stimulus make it, since from the results I can hardly see the stimulus artifacts.

Stimulus artifacts occur when electromagnetic activity related to stimulus presentation is recorded in the EEG, and are largely caused by electromagnetic leakage of the earphone transducers (Akhoun et al , 2008) generating an electromagnetic field picked up by the EEG. In the present study, we have addressed stimulus artifacts on three accounts: 1) Air-tube insert earphones were employed, creating distance between the earphone transducers and the EEG electrodes. 2) The earphone transducers were shielded with grounded metal boxes which has shown to reduce stimulus presentation artefacts (e.g., Akhoun et al , 2008; Riazi and Ferraro, 2008). The audio signal cables were also shielded, with the cable shield connected to the same ground as the metal box. By shielding and grounding the earphone transducers and cables, the electromagnetic fields generated by the stimulus-producing currents meet the conductor material of the shields, where a current is induced, displacing charge inside the conductor to the ground, thereby cancelling the electromagnetic field which thus no longer contaminates the EEG measurement. 3) Model predictors were computed once for the original and once for the sign-inverted speech stimuli. TRFs were computed for both predictors, and then averaged (see e.g. Maddox and Lee, 2018). This approach is inspired by the traditional approach of using repeated short stimuli of alternating polarity, and then averaging across neural responses. We believe that we were able to reduce stimulus presentation artefacts for the continuous speech due to a combination of the above described methods. We have now expanded the relevant section of the Methods as follows.

"Stimulus artifacts occur when electromagnetic activity related to stimulus presentation is recorded in the EEG, and are largely caused by electromagnetic leakage of the headphone transducers and cables (Akhoun et. al. 2008). Here, we employed several methods to reduce stimulus artifacts: 1) Air-tube insert earphones were employed, creating distance between the headphone transducers and the EEG electrodes. 2) The headphone transducers were shielded with grounded metal boxes which has been shown to reduce stimulus presentation artifacts (Akhound et.

al. 2008, Riazi et. al. 2008). The audio signal cables were also shielded, with the cable shield connected to the same ground as the metal box. 3) Model predictors were computed once for the original and once for the sign-inverted speech stimuli. TRFs were computed for both predictors, and then averaged, following prior work (Maddox and Lee 2018). This approach is inspired by the traditional approach of using repeated short stimuli of alternating polarity, and then averaging across neural responses. Further details on predictor and TRF estimation are provided below. Later visual inspection of TRFs confirmed that stimulus artifacts were not present in the estimated TRFs."

New references:

1. Riazi, Mariam, and John A. Ferraro. 'Observations on Mastoid versus Ear Canal Recorded Cochlear Microphonic in Newborns and Adults'. Journal of the American Academy of Audiology 19, no. 1 (2008): 46–55. https://doi.org/10.3766/jaaa.19.1.5.

2. Akhoun, Idrick, Annie Moulin, Arnaud Jeanvoine, Mikael Ménard, François Buret, Christian Vollaire, Riccardo Scorretti, et al. 'Speech Auditory Brainstem Response (Speech ABR) Characteristics Depending on Recording Conditions, and Hearing Status: An Experimental Parametric Study'. Journal of Neuroscience Methods 175, no. 2 (2008): 196–205. https://doi.org/10.1016/j.jneumeth.2008.07.026.

7. The abbreviation should be rigorous. Simple model without adaptation (OSS). I don't think OSS could be the abbr. of this phrase. So do OSSA, ZIL.

We apologize for the confusion, the abbreviations were based on the names of the authors of each model. The function names for each model in the auditory modelling toolbox also have the same names, and hence we decided to use abbreviated versions of these. Therefore, OSS for Osses model, OSSA for Osses model with Adaptation, ZIL for Zilany model. We have now also clarified the terminology in the Methods section.

8. For figure 1, the authors should provide an explanation for the markers in the figures, like what the circles mean? Also, they should give the SD of the TRFs, and so does figure 2.

Figure 2 (previously Fig 1) right panel shows the boxplots over subjects. Here, the circles indicate outlier datapoints (individual values of model fits). We have now clarified this in the figure caption using the phrase "outlier datapoints are marked using circles". We initially did not want to show SD or SEM (standard error of the mean) in the TRF figures, since it would be difficult to see with so many overlapping curves. Even so, we have now shown SEM in a lighter shade in Fig 2 (previously Fig

1). However, it is not possible to show SD or SEM in Fig 3 (previously Fig 2) since each curve is for an individual TRF and not the average.

9. For figure 3, the statistical analyses should be marked out in the figure.

We prefer not to show the statistical analyses in the figure, since statistical tests were performed on only one condition out of the many shown in the figure. Only the SNR at the 32-minute datalength was used for statistical analysis (rightmost group in bottom row of the figure). Adding statistical significance bars might give the impression that all pairwise comparisons were tested and so we have not added these results. However, we have now incorporated a summary of the statistical analysis in the figure caption as follows.

"The wave V SNRs at 32 minutes of data for the OSSA and ZIL were significantly larger than all other predictors (see Results). Crucially, OSSA wave V SNRs were not significantly different to ZIL."

10. For the whole manuscript, there are discussing wave V, I think indeed they are studying ABR? If so, why the put the results for 30ms? If not, what other indexes they used?

The reviewer is correct in noting that the primary index we use is the wave V of the ABR TRF. We agree that plotting TRFs up to 30 ms is unnecessary and have now shortened the time window to show only -10 to 20 ms of the TRFs (in both Fig. 2 and Fig. 3). We believe this highlights the most significant parts of the subcortical TRF.

This works made some efforts on this field but need to provide more comprehensive and detailed results for supporting their statements.

We hope the additions to the text mentioned above, as well as the updated figures have served to clarify our results and support our conclusions.

Reviewer 2

The submitted article compares 5 computational methods for estimating the subcortical TRF based on Wave V of the ABR from EEG during a passive listening task. Each model is sufficiently described in detail, and the results/conclusions indicate that while the most complex method based on a well-known auditory-nerve model (Zilany et al. 2009) was the best predictor of the TRF, it was computationally much higher than a simpler model with adaptation (OSSA), which also performed well. This leads the author to suggest that practical consideration, for example in assistive listening devices, would perhaps benefit from this computational-performance trade off.

The article is free of any glaring issues, though i have a few questions/comments that could be helpful to the reader.

1) the choice of 3 dB threshold for detecting a meaningful wave V is seemingly arbitrary and not discussed. how might the results change (if at all) if this choice were more liberal or conservative?

> The reviewer is correct in noting that the 3 dB threshold is partly arbitrary. However, some threshold needs to be used to quantify the effects of predictor and data-length. 3 dB is often used to denote a reasonable SNR, and also has the intuitive meaning of the signal power being twice the noise power. In our case, TRFs with approximately 3 dB or higher SNR showed visually distinct wave V peaks. Therefore, we decided to use 3 dB as our threshold for a 'clear' wave V peak. Using another threshold may change conclusions on how much data length is needed (as clearly seen in Fig. 4), but any other threshold would be just as arbitrary as 3 dB. We have now included the following caveat in the Methods when defining the SNR metric.
>
> "This threshold of 3 dB, though arbitrary, has the intuitive meaning of the signal power being twice the noise power. Indeed, individual TRFs with more than 3 dB SNR showed visually distinct wave V peaks, confirming that this value was a reasonable threshold for wave V peak detection."

2) it looks like a few subjects (P01, P07, and P08) have very strong TRFs relative to the others in Figure 2. I may have missed it, but how was this taken into account when performing a grand average? That is, were their relative reponses between computational methods in any way over represented in the grand average, or was their a normalization process used?

> No normalization was used when averaging individual TRFs. Given that the amplitude of the wave V peak is an important measure in traditional ABR measurements, we feel that normalizing could lead to biased results. Rather, by not normalizing the TRFs, we allow for meaningful individual differences to be investigated (e.g., in the Fig. 5. amplitude subplots). We agree that this may result in the TRFs of some individuals dominating the average, and this is one of the reasons that we include Fig. 3. (previously Fig. 2) in the manuscript showing all individual TRFs. Furthermore, we do not make any conclusions based on the average, but rather based on the statistical distributions of individual TRF model fits and wave V amplitudes, latencies and SNRs. Finally, our primary metric, the wave V SNR, can be considered as a 'normalized' metric since it compares the wave V to the noise floor for each individual subject.

3) In figure 3, the rightmost panel of the top row (prediction correlations) should be identical to the boxplots in Figure 1, both of which considered the full 32 minutes? It appears that there are slight differences, especially in the outliers, so this was confusing to me.

> We apologize for the confusion, but even though the full 32 minutes were used for both plots, the actual datapoints are slightly different. This is because in Fig. 2 (previously Fig. 1), the true model fits are provided, while in Fig. 4 (previously Fig. 3), the values after subtracting the null model fits from the true model fits are provided. This is also mentioned in the caption for Fig. 4.

4) were tests of statistical significance run on the correlations presented in Fig 4?

> Thank you for the suggestion. We have now added Holm-Bonferroni corrected p-values for the correlations to the subplot titles. All correlations were significant with $p < 0.05$.

5) check the references - some do not contain the publication year, like Picton and Zilany et al.

> We thank the reviewer for this comment. All the references have now been updated accordingly.
